# First report of canine Astrovirus and Sapovirus in Greece, hosting both asymptomatic and gastroenteritis symptomatic dogs

**DOI:** 10.1186/s12985-022-01787-1

**Published:** 2022-03-31

**Authors:** Efthymia Stamelou, Ioannis A. Giantsis, Konstantinos V. Papageorgiou, Evanthia Petridou, Irit Davidson, Zoe S. Polizopοulou, Anna Papa, Spyridon K. Kritas

**Affiliations:** 1 School of Veterinary Medicine, Faculty of Health Sciences, Aristotle University of Thessaloniki, 54124 Thessaloniki, Greece; 2 Department of Animal Science, Faculty of Agricultural Sciences, University of Western Macedonia, 53100 Florina, Greece; 3 Kimron Veterinary Institute, 50250 Bet Dagan, Israel; 4 Laboratory of Microbiology, School of Medicine, Faculty of Health Sciences, Aristotle University of Thessaloniki, 54124 Thessaloniki, Greece

**Keywords:** Astrovirus, Norovirus, Sapovirus, Dog, Canine, Diarrhea, Circulation, Molecular techniques

## Abstract

**Background:**

Astrovirus, Norovirus and Sapovirus are widely distributed viruses in humans and animals worldwide. They have frequently been associated with disease, mainly of gastroenteric nature. In dogs, these viruses have been detected both in symptomatic and asymptomatic animals, mainly of young age.

**Methods:**

In the present epidemiologic study, we investigated the presence of canine Astrovirus (CAstV), canine Norovirus (canine NoV) and canine Sapovirus (Canine SaV) in saliva and stools of 201 domestic dogs originating from throughout Greece, based on two different molecular methods, i.e. conventional and SYBR-Green Real-time RT-PCR. The samples derived from young and adult asymptomatic and symptomatic animals. CAstV was detected in 15/201 (7.5%) and 29/201 (15%) of the examined dogs using conventional RT-PCR and SYBR-Green Real time RT-PCR, **r**espectively.

**Results:**

The prevalence of the virus was higher at healthy dogs, with a slight discrepancy of the two methods on the aspect of age (67% young dogs with the method of conventional RT-PCR, versus 52% adult positive dogs with the method of SYBR-Green Real-time RT-PCR). Canine SaV was detected in 52/201 (23%) of the dogs (mainly young and asymptomatic), with the method of SYBR-Green Real-time RT-PCR only, while canine NoV was not detected in any sample with either of the two methods applied. Sequencing of the CAstV positive samples resulted in the acquisition of one CAstV sequence. Phylogenetic analysis confirmed the results, clustering the CAstV sequence with homologous canine hosting sequences from other countries.

**Conclusions:**

CAstV and Canine SaV were proved to circulate in Greek dogs. SYBR-Green Real time RT-PCR showed greater sensitivity in the detection of these viruses. Additionally, we were able to specify the CAstV strain that circulates in Greece, through phylogenetic analysis. To our knowledge, this is the first epidemiological study of CAstV and canine SaV in dogs in Greece, as well as the first time detected in dogs from Greece.

## Background

The enteropathogenic viruses Astrovirus, Norovirus and Sapovirus may be responsible for the occurrence of diarrhea episodes in various mammals including pigs, cats, mink, humans and dogs, as well as in birds [1–5]. Due to their worldwide distribution, and high transmission capability, these viruses, and particularly Noroviruses (NoVs) and Sapoviruses (SaVs), are considered the most common causative agents of human viral gastroenteritis throughout the globe [2].

Astroviruses are members of the family Astroviridae, which are in general non-enveloped, round-structured, and single-stranded RNA viruses. They are small viruses, of an approximate 28–30 nm diameter, containing a positive ssRNA genome of 6.4–7.7 kb in length [6]. The family Astroviridae is divided into two genera, namely Mamastrovirus, that includes 19 species, and Avastrovirus that includes 3 species [7]. Members of the genus Mamastrovirus infect various mammals, including human [8], bovine [9], feline [10], porcine [11] and mink [12]. Members of the genus Avastrovirus infect mainly avian species, such as chicken, turkey, and duck [13–15]. Mamastrovirus and Avastrovirus genomes incorporate three open reading frames (ORFs) (ORF1a, ORF1b, and ORF2) [16–18]. Infectious viral RNA acts as both genomic and viral mRNA [19, 20]. Worth also mentioning that Astroviruses are estimated to cause approximately 10% of the gastroenteritis cases in children worldwide [21].

Canine Astroviruses (CAstVs) were first detected in 1980, in feces of diarrheic puppies in the USA [22]. Since then, they have been identified in various countries around the world [16, 23–26]. CAstVs are widespread in canine populations and are characterized by high genetic diversity [27]. This feature of CAstV makes diagnosis or future control strategies extremely challenging [28]. CAstV has been reported in the feces of dogs with and without diarrhea [29]. Most studies have shown that the positive rate of CAstV in dogs with symptoms of gastroenteritis is higher than in asymptomatic ones [29, 30] indicating a potential role in canine diarrhea. However, data concerning the clinical significance or association of astrovirus infection with diarrhea or other infectious diseases remains limited.

Noroviruses and Sapoviruses belong in the family Caliciviridae. Both genera constitute non-envelopped, single-stranded, positive-sense RNA viruses of larger size than Astroviruses, i.e. 7.3 to 8.5 kb in size [31]. The Caliciviridae family is further classified into two groups based on their genomic structure [32]. The first group contains Norovirus, Vesivirus and Recovirus and in this group an open reading frame 1 (*ORF1*) is separated from *ORF2* and *ORF3* near the 3’ end whereas an *ORF4* that is comprised within *ORF2* was found in MuNoV, encoding the virulence factor, namely *VF1*. The second group, containing the Sapovirus, is composed by a larger *ORF1* and a standard *ORF2* that is equivalent to the *ORF3* of the Norovirus. Within this group, an *ORF3* is proposed as equivalent to *ORF4*. Based on the capsid gene variation (*VP1* region), these two virus genera are divided into several genogroups and genotypes.

Concerning Norovirus, it is divided into ten genogroups (GI-GX) and 48 genotypes [33]. Three of these genogroups (GI, GII and GIV), have been detected in humans. Sapovirus is more divergent, classified into 19 genogroups (GI-GVIII), of which the four (GI, GII, GIV and GV) have been isolated from humans, and at least 52 genotypes based on complete VP1 sequences [34, 35]. Genetically closely related to human Sapovirus strains apart from human have been identified only in chimpanzee (GI) [36], rodents (GII) [37], California sea lions (GV) [38, 39] and pigs (GV and GVIII) [40, 41]. Canine Sapoviruses (Canine SaVs) belong to the genogroup GXIII [42] whereas Canine Noroviruses (canine NoVs) belong to genogroups GIV and GVI [43].

In the present study, we examined the feces and saliva of 201 dogs, originating from two age groups i.e. young (< 12 months) and adults (≥ 12 months), from different areas around Greece, for the presence of CAstV, canine SaV and canine NoV. The samples derived from both asymptomatic and symptomatic animals, mainly with gastroenteritis symptoms. Two different protocols were applied, the first of which based on a conventional reverse transcription PCR (RT-PCR), while the second on a SYBR-Green Real-Time RT-PCR, in a sensitivity and specificity comparison approach. Results were confirmed by sequencing and phylogenetic analyses. To the best of our knowledge, at least for Astrovirus and Sapovirus, this is the first epidemiological research in dogs in Greece.

## Materials and methods

### Sample collection

**Fig. 1:**
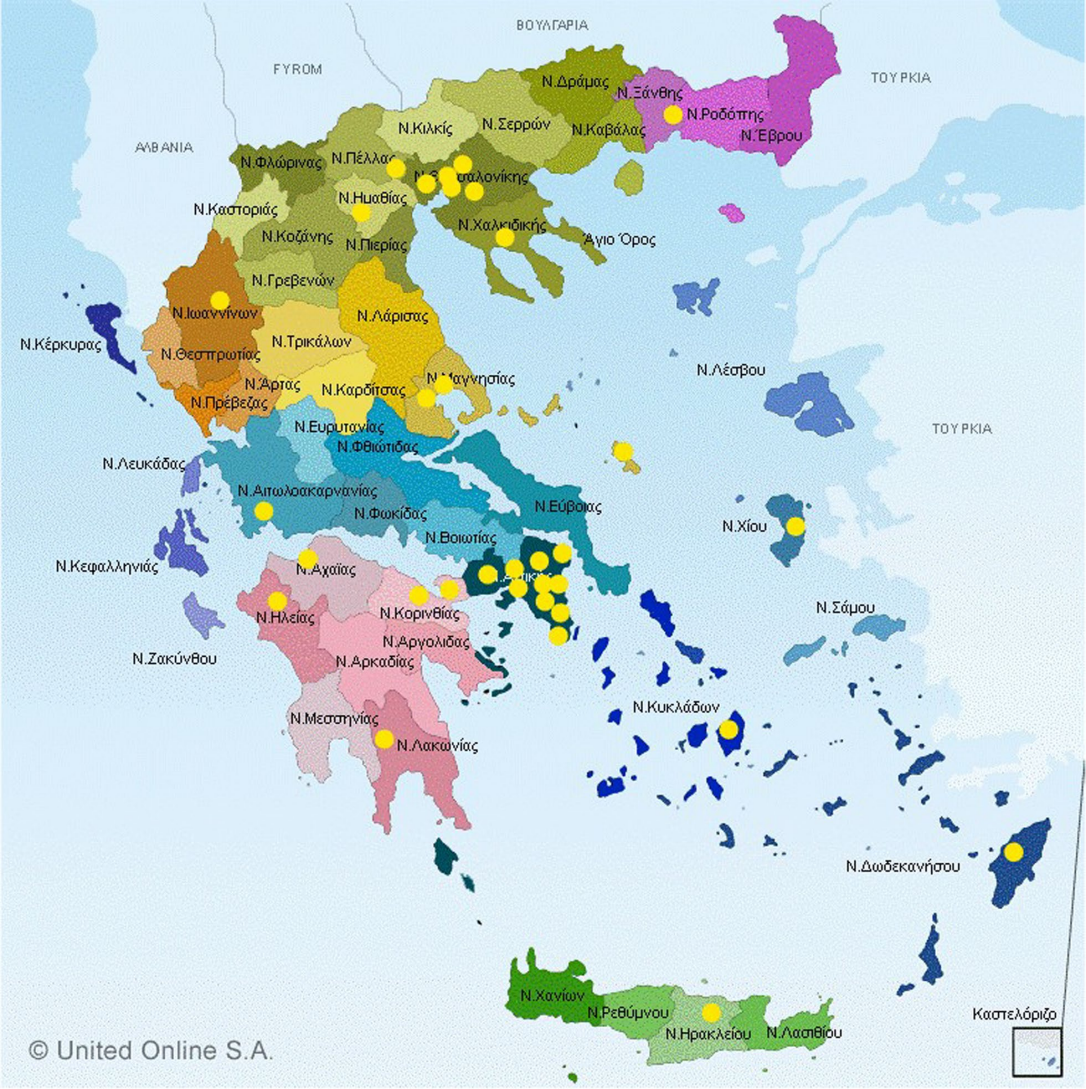
Map of Greece demonstrating the sample collection sites, indicated in the form of yellow dots

In the period between 2017 and 2018, 201 fecal swab samples and 201 saliva samples were collected from 201 domestic dogs throughout Greece (Fig. 1). Thirty-three veterinaries were chosen for the sample collection, whereas the samples were collected by veterinarians from dogs visiting their veterinaries. The samples derived from both symptomatic and asymptomatic dogs of various breeds. The symptomatic animals presented mainly gastroenteritis symptoms, but dogs with other symptoms were also included in the study. The dogs were divided in two age groups: young (< 12 months old) and adult (≥ 12 months old). Two swab samples were obtained from each dog, one saliva sample and one fecal sample. After the sampling, the swab was inserted in a 1,5 ml eppendorf tube, which contained 1 ml of RNAlater (Sigma Aldrich) solution for the stabilization of RNA and transferred to the lab in ice in isothermic boxes. After arriving at the lab, each sample was vortexed for 5 min and centrifugation was performed at 12,000 × g for 10 min. The supernatants were collected, pooled together in pools of 3–5 (which were placed in new sterile 1,5 ml eppendorf tubes) and stored at –80 °C until further processing. In total, 92 pools were created from the original 402 individual samples.

### RNA extraction

Total RNA was extracted from a 200 μl volume pooled sample, using the RNA extraction kit “Cador pathogen kit” (Qiagen, Germany), following the manufacturer’s recommendations. Quality and quantity of the extracted RNA were evaluated in a Spectrophotometer (Eppendorf). Extracted RNA was stored at – 80 °C till further analyses.

### B-actin detection for the affirmation of the integrity of the samples

To confirm the RNA integrity of the samples, a two-step RT-PCR for the detection of b-actin was performed. Actin is the most abundant protein that is present at most eukaryotic cells. Actin gene is a major house-keeping gene, which means that it is a management gene that exists in all animal species, including humans, stably expressed in most of the tissues regardless of the physiological condition. For the detection of b-actin in dogs, primers actin2 (5′-CTA GAA GCA TTT GCG GTG GAC GAT-3′) and actin3 (5′-GCT CCT CCC TGG AGA AGA GCT A-3′) were used [44] in a two-step RT-PCR with the following conditions: Reverse transcription was performed in two mixtures, using the PrimeScript™ RT-PCR (TAKARA, Japan); mixture one contained 1 μl dNTPs (10 mM), 1 μl oligo dtPrimer (2.5 μM), 100–150 ng template RNA and RNAse free water up to a total volume of 10 μl. The mixture was incubated for 5 min at 65 °C and then stored at 4 °C. The second mixture was then prepared, which contained 2 μl 5X PrimeScript Buffer, 0.25 μl RNase inhibitor (40 U/μl), 0.5 μl PrimeScript RTase, RNase free water up to the final volume of 10 μl and 5 μl reaction mixture used for denaturation and annealing from Mixture 1. Mixture 2 was subjected in a thermocycler programmed at 30 °C for 10 min, 50 °C at 30 min and 95 °C for 5 min. In this way, reverse transcribed cDNA was created. PCR for the detection of b-actin was performed in the following way. Mastermix contained 3 μl of 10X PCR Buffer, 0.6 μl of dNTPs (10 mM), 1.5 μl of MgCl2 (50 mM), 0.6 μl of Forward primer (10μΜ), 0.6 μl of Reverse primer (10 μM), 0.3 μl of 5 u/μl Taq polymerase and 18.4 μl Rnase-free H_2_O. Four μl of cDNA were added to the mastermix. The conditions of the PCR were the following: initial PCR activation step at 95 °C for 2 min, followed by 40 cycles of 95 °C for 1 min, 62 °C for 1 min and 72 °C for 1 min and a final extension step of 72 °C for 10 min.

### Conventional PCR

#### Astrovirus

For the detection of canine Astrovirus using conventional PCR, the primers 625F (5′-GTACTATACCRTCTGATTTAATT-3′) and 626R (5′-AGACCAARGTGTCATAGTTCAG-3′) [5] were used. These primers produce an amplicon of 294 bp. The cDNA that was previously generated, was used in conventional PCR. The Mastermix contained 3 μl of PCR Buffer, 0.6 μl of dNTPs, 1.2 μl of Forward primer, 1.2 μl of Reverse primer, 0.36 μl of Taq polymerase (10 units/μl) and 19.64 μl of Rnase-free H_2_O. 4 μl of cDNA were added to the mastermix. The conditions of the PCR were the following: initial PCR activation step at 94 °C for 2 min followed by 40 cycles of 1 min at 95 °C, 1 min at 51 °C and 1 min at 72 °C and a final extension step at 72 °C for 10 min.

#### Norovirus and Sapovirus

For the detection of canine Norovirus and Sapovirus, three different primer pairs were used. The first was the universal primer pair p289-p290(p290: GATTACTCCAAGTGGGACTCCAC-p289: TGACAATGTAATCATCACCATA, [45], which has the ability to simultaneously detect Norovirus and Sapovirus, producing an amplicon of 331 bp for Sapovirus and 318 bp for Norovirus, respectively. This primer pair targets a conserved region of the *RdRp* of Caliciviruses. The second primer pair was HK primer pair for the detection of Norovirus (ΗΚ NORO-F (5′-RHYATTGACCCCTGGATW-3′) and HK NORO-R (5′-AACGCATTCCCHGCMARKA-3′, [46], producing an amplicon of 203 bp. This primer pair targets the most conserved region of HK Norovirus strain VP1protein. As positive control we used HK Norovirus strain, which S. Caddy kindly granted to us from UK integrated into plasmid. The third primer pair was DogSap1F (5′-ACACACGATCCAAATTCACCAA-3′) and DogSap1R (5′-TGCCAGACAGACCTCCAATTG-3′) [47] for the detection of Sapovirus, which produces an amplicon of 150 bp.

For the PCR reaction with primer pair p289-p290, 5 μl of 10 × PCR Buffer, 2 μl of dNTPs, 1 μl of Forward primer, 1 μl of Reverse primer, 0.5 μl of Taq polymerase (10 units/μl) and 36.5 μl of Rnase free H_2_O were added to 4 μl cDNA. The conditions of the PCR were the following: 94 °C for 3 min, followed by 40 cycles of 30 s at 94 °C, 1:20 min at 49 °C, 1 min at 72 °C and a final extension step at 72 °C for 10 min.

For the PCR reaction with the primer pair HK NORO-F-HK NORO-R, 3 μl of 10 × PCR Buffer, 0.6 μl of dNTPs, 1.2 μl of Forward primer, 1.2 μl of Reverse primer, 0.36 μl of Taq polymerase (10 units/μl) and 19.64 μl of Rnase free H_2_O were added to 4 μl cDNA. The conditions of the PCR were the following: 94 °C for 3 min, followed by 40 cycles of 30 s at 94 °C, 1 min at 60 °C and 1 min at 72 °C, and a final extension step of 10 min at 72 °C.

Finally, for the PCR reaction using the primer pair DogSap1F-DogSap1R, 3 μl of 10 × PCR Buffer, 0.6 μl of dNTPs, 1.2 μl of Forward primer, 1.2 μl of Reverse primer, 0.36 μl Taq polymerase and 19.64 μl of Rnase free H2O were added to 4 μl cDNA. The conditions of the PCR were the following: initial activation step at 94 °C for 3 min, followed by 40 cycles of 30 s at 94 °C, 1 min at 60 °C and 1 min at 72 °C and a final extension step of 10 min at 72 °C.

### SYBR-Green Real time RT-PCR

#### Astrovirus

For the detection of canine Astrovirus with the method of Sybr-Green Real time RT-PCR, the primers F2-R2(F2: 5′-TTCCCTGCTTCTGATCAG-3′ and R2: 5′-CTCACTTAGTGTAGGGAGAG-3′) were used [48]. These primers target the Cap gene of canine Astrovirus and produce an amplicon of 126 bp. The Fast Gene IC Green One Step Mix kit was used for the reaction of the Sybr-Green Real time RT-PCR (Nippon Genetics). RT-PCR conditions and melt curve analysis was carried out as described in our previous work [49].

#### Norovirus

For the detection of canine Norovirus with the method of of SYBR-Green Real time RT-PCR, the primers F: GCTGGATGCGGTTCTCTGAC and R: TCATTAGACGCCATCTTCATTCAC were used [46] with RT-PCR reagents and conditions, and melt curve analysis exactly as in Stamelou et al. (2022) [49]. The target sequence is the highly conserved region of the *RdRp.*

#### Sapovirus

The primers DogSap1F-DogSap1R were also used for the Sybr-Green Real time RT-PCR for the detection of canine Sapovirus [47]. Fast Gene IC Green One Step Mix kit was used for the reaction of the Sybr-Green Real time RT-PCR (Nippon Genetics). The consentrations of the reagents and the conditions of the PCR are the same with those in Stamelou et al. [49] for the detection of canine Norovirus.

### Sequencing

Positive Astrovirus or Caliciviruses samples based on the conventional RT-PCR, were purified and bidirectionally sequenced in Eurofins Scientific. The software MEGA-X was utilized for editing of the sequences [50] and the Basic Local Alignment Search Tool (BLAST) was applied for sequence similarity assessment of the derived haplotypes with respective ones derived from the Genbank database (available at http://www.ncbi.nlm.nih.gov/blast/Blast.cgi). The same software was utilized for the construction of the phylogenetic trees applying the neighbor-joining method, validated by 1000 bootstrap iterations.

## Results

**Fig. 2:**
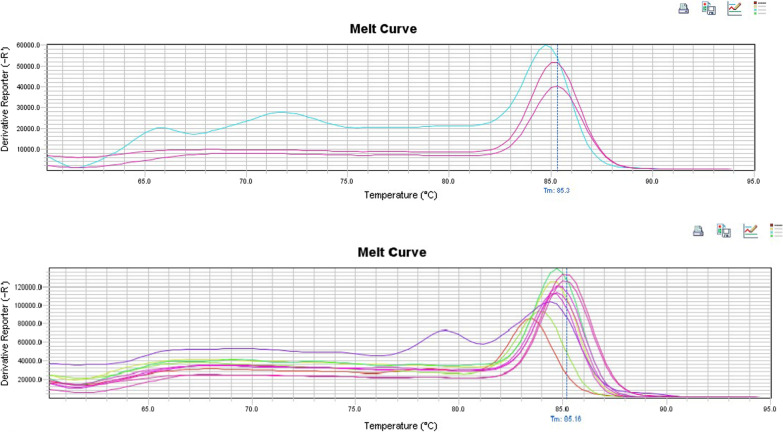
Melting curve profiles of the examined canine samples that were positive to canine Astrovirus. Each peak indicates each analysed amplicon

**Fig. 3:**
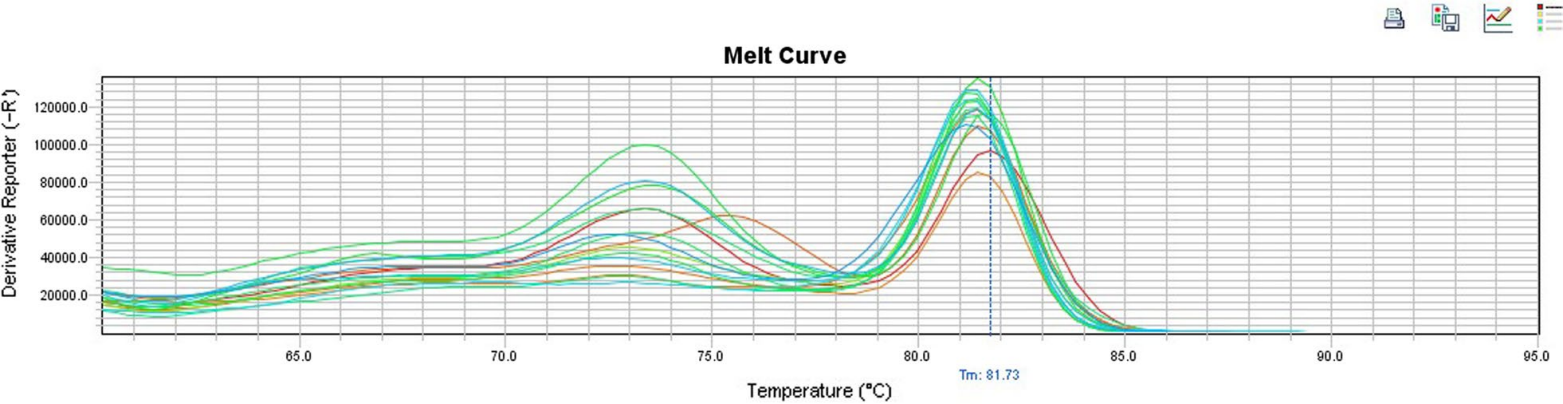
Melting curve profiles of the examined canine samples that were positive to canine Sapovirus. Each peak indicates each analysed amplicon

B-actin gene was successfully amplified in all samples reassuring their integrity. Canine Astrovirus was detected in 7.5% (15/201) of the dogs using the conventional RT-PCR and in 15% (29/201) of the dogs using the SYBR-Green Real time RT-PCR combined with melting curve analysis (Fig. 2). Tm values were 86.5 °C and 81.5 °C for canine Astrovirus and canine Sapovirus respectively. Sixty seven percent (67%) of the dogs that were tested positive in the conventional RT-PCR were young and healthy, while 33% were adult and symptomatic. Regarding the age distribution of the virus with the method of SYBR-Green Real time RT-PCR, 48% of the positive dogs were young and 52% of them were adults. 59% of the dogs in which the virus was detected, were asymptomatic, whereas 41% of them were symptomatic (mainly with gastroenteric symptoms). On the contrary, canine Norovirus was not detected in any of the samples examined neither using the conventional RT-PCR, nor the SYBR-Green Real-time RT-PCR. Canine Sapovirus was detected in 23% (52/201) of the dogs using the SYBR-Green Real-time RT-PCR followed by a melt curve analysis (Fig. 3). Nevertheless, it was not detected in any sample when the conventional RT-PCR was applied. Regarding the age distribution, 52% of the positive dogs were young, while 48% were adult dogs. 56% of the positive dogs were asymptomatic and 44% of them were symptomatic, mainly expressing symptoms of gastroenteritis.

**Fig. 4:**
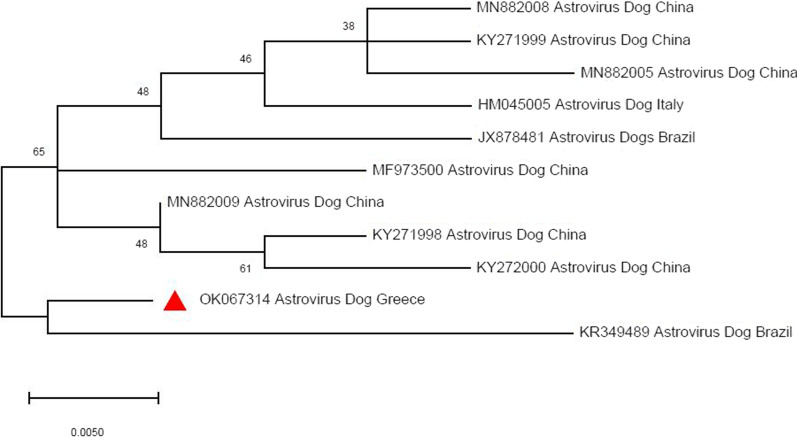
Neighbor joining phylogenetic analysis of nucleotide sequences from the Cap gene (294-bp fragment) of a canine Astrovirus strain detected in this study (indicated with red triangle) in comparison with 10 canine Astrovirus reference sequences. GenBank accession numbers, and country of origin are shown on the branches of the tree

The samples that were positive to canine Astrovirus in the conventional RT-PCR were sent for sequencing. One canine Astrovirus sequence was acquired, and was deposited in the GenBank database under the accession number OK067314. A phylogenetic tree was constructed (Fig. 4); it was shown that the sequence presented 95% similarity with respective canine Astrovirus sequences.

## Discussion

Viruses constitute the main causative agents of gastroenteritis in animals, especially in puppies (< 1 year old) [51]. Canine Astrovirus, canine Norovirus and canine Sapovirus are relatively newly described viruses, which have been associated with gastroenteritis symptoms in dogs but the precise mechanism in disease remains unclear.

In the present study, canine Astrovirus was detected in 7.5% (30/402) of the samples with the method of conventional RT-PCR, and in 15% (60/402) of the samples with the method of SYBR-Green Real time RT-PCR, suggesting that the second has a higher sensitivity. Based on conventional RT-PCR results, the virus was mainly detected in young, healthy dogs (67%), while based on the SYBR-Green RT-PCR results, the virus was detected mainly in adult, healthy dogs. This discrepancy can be probably explained by the fact that more dogs were tested positive with the method of SYBR-Green Real-time RT-PCR, resulting in this alteration, that is further attributed to the nature of each technique, with real time PCR being capable of detecting lower quantities of viral RNA, in terms of higher sensitivity. The melting temperature of samples that were positive to canine Astrovirus with the method of SYBR-Green Real-time RT-PCR was 86.5 °C (Fig. 2). Our findings agree with the results of Wang et al. [48], regarding the melting temperature of the samples in the SYBR-Green Real-time RT-PCR. As far as the nature of the samples is concerned, the virus was detected both in feces and saliva samples with approximately the same proportion. The positive samples were sent for sequencing, which resulted in the acquisition of only one canine Astrovirus haplotype, indicating high levels of genetic homogeneity. The phylogenetic tree created, including various canine Astrovirus haplotypes retrieved from GenBank, showed over 98% genetic similarity with our sequence (Fig. 4), confirming the presence of this virus in the examined samples. As observed in Fig. 4, the canine Astrovirus sequence that acquired in the current study is genetically highly similar with canine Astrovirus sequences from Brazil, China, and Italy, with the sequence of Brazil showing the greatest similarity. This genetic similarity can be interpreted by several reasons, such as travelling in these countries. Dog owners usually travel with their pets. Therefore, it is possible that the virus could have been transferred directly in Greece by a dog that travelled in one of these three countries or indirectly through hosting dogs in intervening countries. The dog that tested positive in the canine Astrovirus sequence that we acquired, belonged to Pressa Canario breed. The country of origin of this breed is Spain. Another possible explanation of the transmission of the virus is the dogs’ shows and dogs’ agility courses that are frequently organized in a lot of countries. As this dog is a thoroughbred dog, it could not be precluded that Astrovirus was transmitted to him in such an event. Moreover, there is a possibility that this dog was purchased by breeders abroad, thus providing another potential of the transmission of the virus. In any case, phylogenetic analysis revealed a pattern of absence of genetic isolation by distance, which is generally in line with the wide trade transports of dog breeds, mainly purebred ones.

On the other hand, Sapovirus was detected in 23% (52/201) of the dogs examined, with the method of SYBR-Green Real-time RT-PCR. It was not detected in any of the samples examined with the method of conventional RT-PCR, with either p289-p290 primer pair or DogSap1F-DogSap1R primer pair. This methodological disagreement has to be taken into consideration in studies targeting the detection of Sapovirus, suggesting using both approaches in an effort to obtain more precise diagnostic results. This result indicates the higher sensitivity of SYBR-Green Real-time RT-PCR compared to the conventional RT-PCR method in this case as well. Canine Sapovirus detection was slightly higher in young, healthy dogs (52% of the dogs that tested positive were young and 56% of them were healthy). We see that both viruses (canine Astrovirus and canine Sapovirus) were mainly detected in asymptomatic dogs. This finding is in consistency with results from other studies, such as Martella et al. [5], where Astrovirus was detected in both symptomatic and asymptomatic dogs. In Zhang et al. [28], canine Astrovirus was also detected both in symptomatic (with symptoms of diarrhoea) and asymptomatic dogs.

Norovirus was not detected in any of the samples examined, either with the method of conventional RT-PCR or SYBR-Green Real-time RT-PCR, although 3 different primer pairs (p289-p290, HK NORO-F-HK NORO-R, F-R) were used for the detection of the virus. Our results agree with the results of Caddy et al. [46], were a SYBR-Green Real-time RT-PCR was performed with primers p289-p290 and another SYBR-based qPCR was performed with HK-NORO-F-HKNORO-R primers for the detection of HK Norovirus (the same strain was used as positive control in our PCR as well). No canine Norovirus positive sample was detected in Caddy et al. study using either primer pair, although antibodies against canine Norovirus were detected in significant levels of the samples examined. A possible explanation for this might be the high genetic variability of canine Norovirus, which may result in the disability of the primers to amplify the virus. Moreover, infection with the virus might be very short-term, thus making the detection with molecular assays very difficult.

## Conclusions

In the present study, we proved the circulation of canine Astrovirus and Sapovirus in dogs throughout Greece. The two viruses were detected both in symptomatic and asymptomatic animals, so their role in disease remains uncertain. The young dogs seem to be those that are mainly affected by the two viruses. We were able to characterize one canine Astrovirus sequence and observe its phylogenetic relationship with other canine Astrovirus sequences from GenBank. Canine norovirus was not detected in the present study. Finally, to our knowledge, this is the first report of canine Astrovirus and Sapovirus in Greece, as well as the first epidemiological study of these viruses in Greece.

## Declarations

### Availability of data and materials

All data generated or analyzed during this study are included within the manuscript as well as in public databases where they have been deposited.
